# Active Oxygen

**Published:** 1875-03

**Authors:** 


### ACTIVE OXYGEN.

The great motor of the human body is oxygen. It is introduced in breathing into the more than five million air-cells of the lungs, and is seized by the red cells of the blood and carried throughout every tissue, to combine with its carbon and hydrogen, thereby producing combustion and extricating heat; and, as oxydation, as it is called—by which is meant the effects resulting from contact with oxygen—is the source of all the functions of the human body, it is evident that without oxygen we could neither think nor act, and life would cease.

It is apparent that if our supply of oxygen is greatly diminished we must suffer in various ways. It is equally evident that the demands which are made upon the atmosphere surrounding us may deprive it of much of this essential fluid. In the vicinity of great factories and foundrys, where noxious exhalations are constantly evolved, the demands made upon this purifying oxydizing element are very great. As a result, the life-supporting properties of the atmosphere are much diminished.

In the best and purest and least contaminated atmosphere, the oxygen contained therein is the most active and potent. It is then known as ozone. In this form it exerts an important purifying effect upon the atmosphere by destroying, through oxydation, and thus neutralizing, all effluvias, which, if not thus acted upon, accumulate until disease is engendered. The ozone is absorbed by these offensive substances, which, in turn, it converts into harmless compounds.

All intelligent readers will have noticed the universal sweetness and freshness of the atmosphere after a thunder-storm. We are apt to declare that the lightning and thunder have purified the air; but we do not consider the method of its action. The air *is* thus purified, but ozone does the purifying work. A product of electrical action is ozone; and even the atmosphere of cities—usually very poor in this vitalizing fluid—is found to contain it abundantly, for an hour after a heavy discharge of thunder.

The power of ozone as a purifying agent may be imagined from the well demonstrated fact, that one part of ozone is adequate to the purification of three million two hundred and forty thousand parts of putrid air. This minute proportion is sufficient to oxydize and burn up all miasmatic exhalations, and to secure pure and wholesome air. If the supply is constant, malaria is impossible. It is nature's great scavenger, disinfecting and cleansing the air, and so securing for those who inhale air thus purified, good blood. It is the one all-powerful destroyer of poisonous exhalations; of atmospheric impurities; of noxious gases; of disease germs; of those poisonous microscopic organisms, which, when inhaled and thus brought in contact with the lungs, and received into the circulation, give rise to what are known as septic or fermentive diseases, such as intermittent fever, or ague, scarlet fever, typhus, cholera, diptheria and all affections of the breathing organs and intestines.

In thousands of instances, physicians advise "a change of air" for patients, when all else seems to fail. Why this is advised, few fully comprehend, or why the change is beneficial. If the facts could be knowr it would be discovered that the good results largely depend upon the amount of ozone existing in the new locality. It is important, then, that physicians should be correctly informed concerning the quantity of this vital principle in the air of various localities, in order that they may give wise counsel.

Fortunately, the tests for ozone in the atmosphere are simple,—so simple that any person can apply them without material cost. A slip of pure white paper, freshly saturated with tincture of gum guaiacum, and at once exposed to the air to be tested, is turned light-blue if ozone exists. This is the method of Fremy; that of Schœnbein is to dissolve one part of iodide of potassium in two hundred parts of water, gently heat the solution, and then stir in gradually ten parts of fine starch. Clean paper is then dipped in the solution and dried in the air. The moment it is dry it must be closely wrapped in tinfoil or enclosed in a glass-stoppered bottle. In using the paper, it must be moistened in water and exposed to the air, but not to any strong wind. If ozone is present, the paper will become blue, and the more ozone exists the deeper and darker will the blue tint become.

It would be a wise act if all medical men, in all parts of the country, would have this test-paper on hand, and constantly expose it, at all seasons and under all changes of temperature, keeping accurate data of the result. As no doubt can possibly exist as to the superior healthfulness of an atmosphere rich in ozone—providing no other cause of disease exists (by which we refer especially to drainage and water supply), the records which we suggest would do more toward settling the question of healthy and unhealthy localities than all the mere opinions which could possibly be uttered. If these tests are widely applied, many inhabited sections will be found to lack this essential principle, while other regions will be shown to possess it abundantly. As science advances, the localities thus proven unhealthy will be abandoned by families, and those abundantly supplied with this active principle of life will be selected by the thoughtful for homes.

---

Lazy Persons die young. It is the active in body and brain who live to extreme old age, as a rule. It is abundantly proven that exercise of the mind invigorates its bodily receptacle, even when that exercise is carried to an apparently extreme point. The brain, the reservoir of nervous energy to the rest of the system, increases in volume and vigor by use, just as the arm of the blacksmith or the leg of the dancer gains in muscular development. The general system benefits by the enhanced brain-power, and greater vitality and longevity is the result. Work by method and on system, even when severe, is not only quite compatible with prolonged life, but is actually conducive to it, while the torpor of idleness or the excitements of fitful effort are the sure precursors of mental and physical degeneration. This is a useful doctrine to preach, and still more useful to practice.

---

A little trade with profit is better than a great concern at a loss; a small fire that warms you is better than a large fire that burns you.

